# In the Age of Synthetic Biology, Will Antimicrobial Peptides be the Next Generation of Antibiotics?

**DOI:** 10.3390/antibiotics9080484

**Published:** 2020-08-06

**Authors:** Félix Jaumaux, Luz P. Gómez de Cadiñanos, Philippe Gabant

**Affiliations:** Syngulon, Rue du Bois Saint-Jean 15/1, 4102 Seraing, Belgium; fjaumaux@syngulon.com (F.J.); lperezgdc@syngulon.com (L.P.G.d.C.)

**Keywords:** synthetic biology, bacteriocins, antibiotics, antimicrobials

## Abstract

Antibiotics have changed human health and revolutionised medical practice since the Second World War. Today, the use of antibiotics is increasingly limited by the rise of antimicrobial-resistant strains. Additionally, broad-spectrum antibiotic activity is not adapted to maintaining a balanced microbiome essential for human health. Targeted antimicrobials could overcome these two drawbacks. Although the rational design of targeted antimicrobial molecules presents a formidable challenge, in nature, targeted genetically encoded killing molecules are used by microbes in their natural ecosystems. The use of a synthetic biology approach allows the harnessing of these natural functions. In this commentary article we illustrate the potential of applying synthetic biology towards bacteriocins to design a new generation of antimicrobials.

## 1. Introduction

Antimicrobials are compounds that kill or inhibit the growth of microorganisms. It could be argued that the availability of antimicrobials, mainly in the form of antibiotics, has been the most significant scientific achievement of the twentieth century, improving human quality of life and increasing life expectancy. However, the benefits provided by non-toxic broad-spectrum antibiotics are being overcome by the rise of antibiotic resistance. The World Health Organization has considered antibiotic resistance to be a major threat, as it has the potential to cause 10 million deaths per year by 2050 [1].

Modern lifestyles and the global economy demand the increase of our anti-infective arsenal to keep our sanitary environment, and thus our health, under control. Antimicrobial resistance (AMR) is an ecological problem characterised by complex interactions involving diverse microbial populations affecting the health of humans, animals, and the environment. Better management of the AMR problem includes targeted antimicrobials that have been reported as alternatives to broad-spectrum antibiotics, such as CRISPR-Cas (Clustered Regularly Interspaced Short Palindromic Repeats-associated protein) antimicrobials, phage therapy, local release of toxins, and use of suicidal conjugative plasmid systems and bacteriocins. Bacteriocins are a diverse group of natural ribosomally synthesised antimicrobial peptides produced by a wide range of bacteria that play an important role in mediating competition within bacterial communities. Thus, the diversity of bacteriocins collectively provides a broad spectrum of activity. Thanks to the vast amount of genomic data available today, the use of bioinformatics in combination with synthetic biology can serve to identify and produce novel antimicrobials through mining of genomic and metagenomic sequencing data for biosynthetic gene clusters (BGCs) followed by engineered production of the antibacterial gene products [2]. 

With this commentary article, we would like to highlight some of the existing alternatives to broad-spectrum antibiotics facilitated by the rise of synthetic biology. We start by discussing the AMR problem and the importance of the microbiome for human health, providing examples of current research on such interactions and the need for narrow spectrum antimicrobials. Then, the use of bacteriocins, academic sleeping beauties, as a possible solution to AMR is introduced, describing the challenges of turning these natural genes into technological parts of a formulated product in our human therapeutic arsenal. We continue by identifying synthetic biology as a tool for finding alternatives to antibiotics and tackling the clear need for the improvement of diagnostic clinical microbiology. Finally, we conclude by identifying other options, such as bacteriophages or futuristic probiotics, and the challenges that need to be overcome for their implementation. We also present the PARAGEN collection, to our knowledge the first physical collection of bacteriocins that represents a reservoir of “ready-to use” antimicrobials. All these approaches have in common that basic knowledge of biology, including ecology, is required at the molecular level to develop these new therapeutic avenues. 

## 2. Discussion

### 2.1. The Antibiotic Resistance Problem and the Importance of the Microbiome

Worldwide, we are relying more heavily on antibiotics to ensure our medical, nutritional, and economic security while simultaneously causing the decline of their usefulness through overuse and improper use. Most of the antibiotics to treat bacterial infections are active against a broad range of species. This ability to treat an infection early, without the need of identifying the causative agent has resulted in the saving of countless lives and enormous benefits for human health but there are also significant drawbacks. Broad-spectrum antibiotics, through their non-targeted killing activity, disbalance microbiomes. They keep selective pressure on non-medically relevant bacteria to maintain resistance genes. Consequently, microbiomes both in the external environment and within the human body are considered to be genetic reservoirs that could be mobilised via horizontal gene transfer, giving rise to antimicrobial resistance (AMR) [3,4,5]. AMR is an ecological problem characterised by complex interactions involving microbes, humans, animals, and the environment. This vision of the AMR problem as a complex interconnection between microbes and our planet has resulted in the One Health concept. One Health is defined by the WHO and others as “an approach to designing and implementing programs, policies, legislation and research in which multiple sectors communicate and work together to achieve better public health outcomes” [6,7]. 

For decades, the most well-studied microbes have been disease-causing agents, with this focus likely due to their negative health effects. However, an increased susceptibility to infectious disease is also part of the diverse array of human health problems associated with the disruption of the microbiome [8,9] as well as metabolic disorders [10], irritable bowel syndrome [11], and immune disorders such as allergies [12]. 

A well-studied example of microbial dysbiosis (imbalance in the composition and function of microbes) involves the intestinal disorder caused by sudden proliferation of latent *Clostridium difficile* bacteria that are normally held in check by other gut microbes as a result of removal of the suppressing microbes due to broad-spectrum antibiotic treatment [13]. It has been shown that faecal microbial transplantation for severe cases of recurrent diarrhoea caused by antibiotic-resistant *C. difficile* infection is efficacious in approximately 90% of affected patients, demonstrating that healthy gut microbiota can reproducibly correct a severe and specific microbial dysbiosis [14]. 

There is an increasing prevalence of pathogens that harbour acquired resistance determinants. One such pathogen is the highly adaptable bacterium *Staphylococcus aureus*. Serious staphylococcal infections are common in both community and hospital settings, causing skin, wound, bloodstream and other types of infections [15]. This microorganism became resistant to penicillin only two years after large-scale production of this antibiotic began in 1942. Likewise, methicillin was introduced in 1960 and methicillin-resistant *Staphylococcus aureus* (MRSA) strains were observed in 1962 [16,17]. This led to the development of vancomycin in 1972, and vancomycin-resistant enterococci (VRE) emerged in 1988 [18]. Lately, although vancomycin has remained as a first-line drug for the treatment of MRSA infections, *S. aureus* isolates with complete resistance to it have emerged. It has been shown that vancomycin-resistant *S. aureus* (VRSA) is mediated by a gene cluster transferred from VRE [19]. It appears that *S. aureus* is highly capable of reprograming its genome to diminish the effect of conventional antibiotics, making it necessary to identify new antimicrobials to fight such pathogens.

Therefore, there is a clear need for the development of antimicrobials to overcome bacterial infections that do not select for cross-resistance in non-targeted pathogens and that cause zero or reduced collateral damage to the host microbiome. Such agents may not substitute prophylactic therapy, or initial broad-spectrum therapy in the case of patients under life-threatening conditions, but following the identification of the causative pathogen, it would be beneficial to switch to a treatment with narrow-spectrum antimicrobials. 

### 2.2. Bacteriocins: Academic Sleeping Beauties

Over the last few decades, hundreds of antimicrobial peptides (AMPs) have been identified in humans, animals, plants, bacteria, and fungi, but relatively few proceed to clinical trials. Although more than 1500 natural AMPs have been isolated and another 3000 AMPs have been designed [20], only four non-linear AMPs have been approved for clinical use, and twenty AMPs are in clinical trials, most of which target skin infections caused by Gram-positive bacteria [21]. 

Among the large variety of AMPs, there is a subgroup known as bacteriocins, which were first discovered in 1925 [22]. These antimicrobials are commonly defined as ribosomally synthesised peptides produced by different bacteria that can kill or inhibit other microorganisms [23,24]. Bacteriocins exhibit strong activity against their target strains, often in the nanomolar range, thus being in some cases more potent than their antibiotic counterparts [25,26]. Interestingly, most of these peptides are only active against a narrow spectrum of bacteria closely related to the producer. For a long time, this narrow inhibition spectrum was considered a major bottleneck [27]. However, today the ability to target specific bacteria instead of the whole microbiome is what gives bacteriocins the great potential to substitute for other antimicrobial compounds or to be combined with antibiotics.

Bacteriocins are a heterogenous group with a wide range of sizes, structures, modes of action, activity spectra, and target cell receptors. Because of the huge number of reported bacteriocins and constant new discoveries regarding their structures and/or mode(s) of action, their classification undergoes continuous modification. Nevertheless, they may be broadly divided into class I (post-translationally modified), the most naturally occurring bacteriocins, class II (heat-stable, <10 kDa, unmodified), and class III (thermo-labile, >10 kDa) groups [28]. Based on their modifications, these bacteriocin classes are further subdivided. The unmodified (class II) bacteriocins can be divided into four groups according to the classification by Álvarez-Sieiro et al. (2016) [28]. These subclasses are comprised of peptides that contain a YGNGVXC motif (in which X represents any amino acid; the class IIa peptides); two peptide bacteriocins (class IIb peptides); leaderless bacteriocins (class IIc peptides); and unmodified, linear, non-pediocin-like, single-peptide bacteriocins that do not belong to other subclasses (class IId peptides).

It is believed that more than 99% of bacteria produce at least one bacteriocin, with most of them not yet identified [29]. With such variety of producing strains and as the bacteriocins are such a heterogeneous group, it is presumable that the different classes of bacteriocins exert their activity through diverse mechanisms against Gram-positive and Gram-negative bacteria. Nevertheless, it has been shown that most bacteriocins are membrane-active peptides, hence they kill sensitive bacteria by membrane disruption after selective interaction with specific membrane receptors [30,31,32,33,34]. This mode of action is different from that of most antibiotics, which often act as enzyme inhibitors [35].

As mentioned earlier, the broad variety of existing bacteriocins relies on their origins, complexities of production, and mechanisms of action. Bacteriocins are usually synthesised as biologically inactive pre-peptides that include an N-terminal leader peptide attached to the C-terminal pro-peptide [36,37]. Their production could be lethal to the producer strain if specific protection systems are not employed, hence the mechanism of immunity of bacteriocin-producing bacteria makes a distinction between self-produced bacteriocins and bacteriocins produced by other microorganisms. The protection can be promoted by a specific protein and/or the conveyor system [36,38,39]. While there is an increasing interest in bacteriocins produced by Gram-negative bacteria, most application-oriented research is focused on those produced by Gram-positive microorganisms, mostly lactic acid bacteria (LAB). For centuries, LAB have been used for food fermentation and thus are considered safe microorganisms for an intended use (GRAS, Generally Recognized as Safe) [40]. This safe status of the producing organism makes LAB bacteriocins particularly interesting as they are commonly found in foods [41,42] and the gastrointestinal tract of humans and animals [43,44]. Also, LAB bacteriocins have some positive attributes that enhance their usefulness, such as being tolerant to high thermal stress and active over a wide pH range. For many decades, antimicrobial peptides have been used as food preservatives as they are colourless, odourless, and tasteless, with the bacteriocin nisin (E234) being the most representative example [45,46].

In addition to their use as natural food preservatives, these peptides have the potential for use in clinical settings because of their ability to neutralise different pathogens, including MDR pathogens such as the methicillin-resistant *Staphylococcus aureus* (MRSA) strain and vancomycin-resistant *Enterococcus faecalis* (VRE) [45,47,48]. They are presumed to actively participate in gastrointestinal host defence mechanisms by inhibiting one ecosystem, encouraging another, and offering a competitive advantage to bacteria in the intestinal tract [49]. A recent report by Kim et al. [50] describes the ability of gut commensals to increase the resistance of the host to vancomycin-resistant *Enterococcus faecium* (VREf). The authors report that, in a four-strain cocktail, a nisin A variant producer was responsible for reduced colonisation by VRE. Furthermore, they demonstrated the potential of bacteriocins as antibiotic alternatives by finding a direct correlation between the amount of the bacteriocin gene and VRE reduction in germ-free mice containing patient faeces [50].

Despite the huge potential of bacteriocins in a broad range of applications, only two are commercially available for food safety applications: nisin and carnocyclin A. Nisin is a bacteriocin from *Lactococcus lactis* produced worldwide as a concentrated fermented powder or otherwise (e.g., Nisaplin, marketed by Dupont, Delaware, USA; Nisin Vega marketed by VEGA, Zhejiang, China). It exhibits antimicrobial activity against *Clostridium botulinum*, *Listeria monocytogenes,* and *Staphylococcus aureus* among other Gram-positive bacteria. Nisin has been approved as a safe food additive by the Joint Food and Agriculture Organization/World Health Organization (FAO/WHO) since 1969 [51], it has been on the European food additives list since the early 1980s as E234 [52], and it has had GRAS status from the FDA since the late 1980s [53]. It is licensed as a biopreservative in over 50 countries [27]. Carnocyclin A is a potent circular bacteriocin produced by *Carnobacterium maltaromaticum* that has been approved as a biopreservative against *L. monocytogenes* in the USA and Canada [54]. 

Although there is a clear interest by research groups in exploring the full potential of bacteriocins, application of their potential remains to be explored. Bacteriocin production is not an easy task and is often considered a bottleneck for their use at an industrial scale [55,56,57]. To our knowledge, biomedical applications of bacteriocins have not been developed to any significant extent. This might be due the lack of cytotoxicity testing of many bacteriocins, limiting the assessment of their therapeutic usefulness [58]. Also, bacteriocins are susceptible to degradation by proteolytic enzymes and are hence expected to have lower in vivo stability and half-life than antibiotics [59]. Issues also arise regarding their solubility, as the long and/or hyper-hydrophobic peptide design can self-aggregate, obstructing the elongation steps [60]. Another drawback is that, as for antibiotics, acquired resistance may appear, even if this phenomenon appears to be minimal in comparison with antibiotics [61,62].

For the development of applications of any protein it is essential to understand its structure-function relationship. Examples of AMP and cell membrane structure to understand the AMP mechanism of action include the work done on two-component bacteriocin plantaricin EF [63], leaderless unmodified bacteriocins lacticin Q and aureocin A53 [64], and the LL-37 AMP [65]. These type of studies are important for molecule improvement, decreasing the likelihood of resistance, facilitating the regulatory process, and even for developing design strategies to create AMPs which do not exist in nature [66].

**Figure 1:**
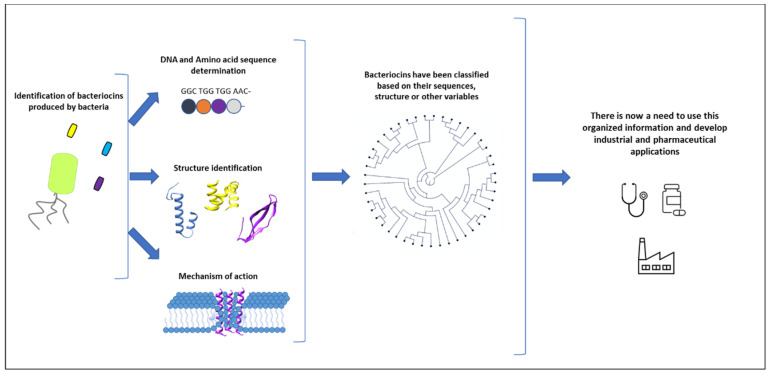
Numerous bacteriocins have been studied by various research groups. When a new bacteriocin is identified, the key elements to determine are its sequence, its three-dimensional structure, and its mechanism of action. Different classifications have been proposed based on this information. However, only a few applications have been explored and brought to market so far. There is now a need to use this organised information to develop new industrial and pharmaceutical applications.

The scientific community has worked and continues to work to isolate new bacteriocins and to study these relationships. This knowledge can today be mobilised using synthetic biology tools to generate and produce new antimicrobial compounds (Figure 1).

### 2.3. Synthetic Biology: Towards a New Generation of Antimicrobials

For many years, the availability of broad-spectrum antibiotics has enabled the treatment of bacterial infections without the need for a specific diagnosis, contributing not only to AMR but also overlooking the importance of diagnostic clinical microbiology [67,68]. The development of diagnostic tests with the necessary speed, accuracy, and sensitivity to rapidly identify a specific pathogen has not been widely pursued [68] and is vital for solving the AMR crisis.

Modern medicine is currently shifting towards a more personalised and customised treatment model that utilises each patient’s disease-related genetic information. However, because understanding of the relationship between genotypes and drug pharmacokinetics is limited [69], optimising the timing, location, and dosage of the administered drug remains problematic. An example of this so called “new medicine” is what has been defined as theranostics [70]. This new medical approach combines specific targeted therapy based on specific targeted diagnostic tests using nanoscience to unite diagnostic and therapeutic applications to form a single agent, allowing for diagnosis, drug delivery, and treatment response monitoring. 

Among the available methods for the precise design of antimicrobials, computational and synthetic biology tools have the most promise. Synthetic biology is a rapidly developing research field resulting in new techniques for the design of genetically modified organisms (GMOs) [71]. By applying engineering principles to molecular biology, synthetic biologists have developed platforms that improve upon and perhaps supplant traditional broad-spectrum antibiotics [72]. This field of research is well positioned to address the challenges in developing new antimicrobials as well as targeted therapies and disease diagnostic tools [70,73]. 

There have been several examples of gene mutation, deletion, replacement, and introduction into natural product-producing bacteria that have generated new compounds, an approach termed combinatorial biosynthesis [74]. Molecular diversification in bioengineering approaches becomes much easier if the antimicrobial molecule is a gene product itself rather than the catalytic result of several gene products whose engineering requires careful coordination [75]. An example of a gene-encoded natural product is the diverse group of ribosomally produced and post-translationally modified peptides (RiPPs), in which bacteriocins are included [76]. In this case, the work by Schmitt et al. 2019 [75] showed that the application of DNA synthesis and modification allows direct synthesis of highly diverse peptides that are then further modified with the functionally important post-translational modification (PTM) machinery.

One area of research that was largely abandoned once penicillin and other potent antibiotics appeared and that has regained traction is that of bacteriophage-based therapeutics. Bacteriophages (phages) are bacteria-specific viruses that insert their genetic information into a host microbe upon infection, taking over essential cellular functions and in some cases inducing microbial lysis [77]. Phages are obligate intracellular parasites, mainly replicating via the lytic or lysogenic lifecycle. The fundamental process behind phage therapy is exclusively the lytic lifecycle with an endpoint resulting in death of the host cell and release of lytic phages [27]. Due to the narrow host range of phages (strain specific in most cases), cocktails consisting of two or more phages can be used to broaden the antimicrobial spectrum and reduce the risk of phage resistance. Phages’ high target-specific nature is precisely what makes them ideal vectors for diagnostic applications [78]. These phage-based biosensors include the combination of complete phages or their constituents, which can be converted to electrical, colorimetric, fluorescent, or luminescent signals and have shown to be cheap, fast, sensitive, selective, and specific tools for detecting bacteria [79]. The revival of phages as therapeutic agents will, such as any new approach, face the difficult challenge of the regulatory barrier. One specific challenge with phages is that they are self-replicating genetically encoded biological entities, thus it will be necessary to take this specificity into account. The use of synthetic biology could overcome this challenge by remastering the genetic code of phages to control their biological properties.

Another potential solution for tackling the AMR problem is based on advances made in harnessing the CRISPR/Cas (Clustered Regularly Interspaced Short Palindromic Repeats/CRISPR associated genes) system for antimicrobial therapeutics [80]. CRISPR-Cas systems have been developed to selectively kill bacterial pathogens or remove AMR genes from bacterial populations, thus re-sensitizing bacteria to antibiotics [81]. The use of synthetic phages or conjugative plasmids has emerged as a powerful vector for CRISPR-Cas delivery to specific groups of bacteria within a mixed population.

As mentioned above, the accelerating pace of AMP discovery has not yet translated into broad clinical trial success. Some of the limitations attributed to AMPs are their poor stability with a short half-life, more or fewer toxic side effects, and the potential for haemolytic activity [82]. Another factor that needs to be addressed is that narrow-spectrum AMPs have very specific applications, hence the market size for them will be more limited in comparison to their antibiotic counterparts [68]. 

An alternative approach to be considered is engineering microbes for therapy in the form of synthetic probiotics, which can lead to the controlled release of these microbial strains providing localised therapeutics under specific conditions. There are many examples in the literature of probiotic strains that have been engineered to act as drug delivery vectors at the site of infection [72,83]. However, there are still multiple challenges in the clinical application of engineered probiotics, such as stable long-term colonization of the niche [84]. In natural florae, bacteria exchange genetic information via plasmid conjugation. The mobilisation of the plasmidome (plasmid-encoded genome) could be a way to specifically fight some individuals in a microbial population. Recently, López-Igual and collaborators [85] designed an elegant application of plasmid conjugation by using a sophisticated suicidal cassette specifically killing targeted bacteria in a population [85]. This application exemplifies how synthetic biology is progressively more applicable and can be used to mobilize different natural artefacts to control microbial populations. 

### 2.4. PARAGEN 

There is extensive scientific knowledge on antimicrobials, and the increasing availability of genomic data has changed the way we identify and study such molecules, such as bacteriocins, in communities. From 2004 to 2015, 429 scientific articles and 245 patents were published or granted related to bacteriocins, and these numbers keep increasing to almost one bacteriocin-related publication per day [86]. This information can now be analysed with an engineering mind set.

Traditionally, large-scale engineering of natural products to obtain improved bioactive molecules has suffered from the lack of efficient systems to explore the antimicrobial activity of large sets of variants at high speed. Today this bottleneck has become obsolete and processing vast amounts of genomic data to search for antimicrobial operons and genes is a relatively easy task thanks to bioinformatic screening tools such as BAGEL [87] and antiSMASH [88].

However, the vast amount of information increases the difficulty in choosing the right bacteriocin for a specific application. Furthermore, in many cases, mutations within genes or tight transcriptional regulation could prevent cells from producing these antimicrobials. In those cases, it might look as if those bacteriocin genes are inactive antimicrobial relics which are unlikely to play an active role, meaning that strains harbouring potentially useful bacteriocins remain uncharacterised due to a lack of in vitro production [89]. Another limitation of the widespread utilisation of bacteriocins has been that research into these peptides is performed by different research groups, and no centralised collection of genes that would allow transitional screening and comparison of their activities and properties has yet been built. 

To overcome these issues, we recently described the first collection of synthetic bacteriocin genes, the PARAGEN collection. This collection is meant to be a repository and arsenal of bacteriocins useful against a wide array of bacteria of medical and industrial importance [90]. Unlike the above-mentioned in silico databases, PARAGEN is a physical collection of bacteriocin genes designed in a standardised format allowing rapid production of peptides available for both academic and industrial researchers. It is a dynamic collection that is continuously expanding and since its first publication, with approximately 130 peptides representing most bacteriocin classes, it has now more than doubled, offering researchers a valuable tool to choose and select the right bacteriocin from our collection tailored for each specific application. 

**Figure 2:**
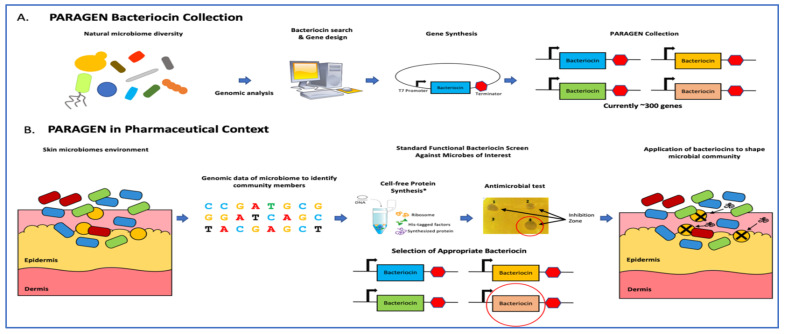
Application of narrow-spectrum antimicrobial peptides to shape microbial populations. (**A**) The PARAGEN collection is a standardised collection of bacteriocins previously described in Gabant and Borrero [90]. This collection was built by exploring bacterial genetic information found in the natural world as well as online databases and gathering bacteriocin-encoding genes in a standardised expression format. Many of these peptides are narrow-spectrum, allowing for control against invasive bacteria without the use of chemical antibiotics. This allows the building of a “genetic firewall” to maintain an optimal production environment. (**B**) In the case of skin contamination, metagenomic analysis identifies the invasive microbe(s). Bacteriocins from the PARAGEN collection with activity against the relevant microbe are identified either through prior knowledge or a standardised functional screen and applied to the contaminated environment to restore the microbial balance.

With Figure 2 we would like to illustrate an example of a potential application of the PARAGEN collection. Recent advances in diagnostic technology allowed us to gain a better understanding of the importance of a healthy skin microbiome to limit skin infections. It has been shown that *Staphylococcus aureus* is responsible for severe skin and deep tissue infections, whereas *Staphylococcus epidermis* plays an important role in maintaining skin health by restricting the growth of *S. aureus* [91,92]. This finding demonstrates the need for the development of new compounds able to selectively kill *S. aureus* while not harming the beneficial microbiome species, especially *S. epidermidis*. This problem could be solved using one or more bacteriocins (Figure 2B).

## 3. Conclusions

The development of targeted antimicrobials is on the agenda for shaping both medical and industrial microbiomes. In human health, urgency is high due to the pressure that AMR is placing on our current drug pipeline. In nature, microbial communities have developed mechanisms to control their ecological balance, allowing constant evolution. These molecular mechanisms have been polished by evolution and are thus very efficient. During the two last decades, genomic approaches have allowed the description of these mechanisms at the genetic level for the first time, allowing the development of rapid and robust diagnoses that are in constant evolution. The possibility of synthetizing genetic codes and gaining faster access to the detailed molecular mechanisms of action of these natural circuits has allowed researchers to apply these mechanisms to produce new compounds able to specifically target deleterious bacteria in a given microbiota. In this short review we have illustrated some of these works, paving the way to a mobilisation of different natural functions by synthetic biology. These works are the first steps in the development of new therapies that will feed our current stock of therapeutic compounds. Basic research to understand bacterial adaptative plasticity will remain essential in order to allow modern medicine to continue controlling infectious diseases and maintain living standards achieved thanks to the use of antibiotics.
